# Method of Manufacturing Structural, Optically Transparent Glass Fiber-Reinforced Polymers (tGFRP) Using Infusion Techniques with Epoxy Resin Systems and E-Glass Fabrics

**DOI:** 10.3390/polym15092183

**Published:** 2023-05-04

**Authors:** Klaus Heudorfer, Johannes Bauer, Yavuz Caydamli, Bruno Gompf, Jens Take, Michael R. Buchmeiser, Peter Middendorf

**Affiliations:** 1 Institute of Aircraft Design (IFB), University of Stuttgart, Pfaffenwaldring 31, D-70569 Stuttgart, Germany; heudorfer@ifb.uni-stuttgart.de (K.H.); bauer@ifb.uni-stuttgart.de (J.B.); take@ifb.uni-stuttgart.de (J.T.); 2 Institute of Polymer Chemistry (IPOC), University of Stuttgart, Pfaffenwaldring 55, D-70569 Stuttgart, Germany; yavuz.caydamli@ipoc.uni-stuttgart.de; 3 German Institutes of Textile and Fiber Research (DITF), Körschtalstr 26, D-73770 Denkendorf, Germany; 4 1st Physics Institute, University of Stuttgart, Pfaffenwaldring 57, D-70569 Stuttgart, Germany; b.gompf@pi.uni-stuttgart.de

**Keywords:** RTM mold design, glass fiber-reinforced polymer (GFRP), epoxy, thermoset, E-glass, transparent, dispersion curves, flexural properties, thermal properties, surface quality

## Abstract

Recently, fiber-reinforced, epoxy-based, optically transparent composites were successfully produced using resin transfer molding (RTM) techniques. Generally, the production of structural, optically transparent composites is challenging since it requires the combination of a very smooth mold surface with a sufficient control of resin flow that leads to no visible voids. Furthermore, it requires a minimum deviation of the refractive indices (RIs) of the matrix polymer and the reinforcement fibers. Here, a new mold design is described and three plates of optically transparent glass fiber-reinforced polymers (tGFRP) with reproducible properties as well as high fiber volume fractions were produced using the RTM process and in situ polymerization of an epoxy resin system enclosing E-glass fiber textiles. Their mechanical (flexural), microstructural (fiber volume fraction, surface roughness, etc.), thermal (DSC, TGA, etc.), and optical (dispersion curves of glass fibers and polymer as well as transmission over visible spectra curves of the tGFRP at varying tempering states) properties were evaluated. The research showed improved surface quality and good transmission data for samples manufactured by a new Optical-RTM setup compared to a standard RTM mold. The maximum transmission was reported to be ≈74%. In addition, no detectable voids were found in these samples. Furthermore, a flexural modulus of 23.49 ± 0.64 GPa was achieved for the Optical-RTM samples having a fiber volume fraction of ≈42%.

## 1. Introduction

Fiber-reinforced polymer composites (FRP) are lightweight and strong materials compared to their alternatives in the universe of materials. With the stiffness of the fibers, the plasticity of the polymer matrix, and the architecture of textiles, a wide variety of composites can be engineered. By adding optical transparency, many applications are conceivable, such as fiber-reinforced windows, canopies for aircraft, transparent and load-bearing elements in architecture as well as safety glass for ballistic armor or visors [1,2,3]. FRPs offer a great variety of benefits such as high specific strength and stiffness in the fiber direction as well as good durability/fatigue properties and high corrosion resistance [4,5,6]. Especially, glass fiber-reinforced polymers (GFRP) show great advantages such as a high elongation at break in comparison to carbon fiber-reinforced polymers (CFRP), and a lower price compared to most other CFRPs [4,5,6,7]. Nevertheless, some challenges need to be overcome to increase the adoption of FRPs, such as their lower mechanical strength and stiffness at elevated temperatures due to the exceedance of the glass transition temperature of the matrix material and the low mechanical properties perpendicular to the fiber direction of unidirectional composites [4,7].

As outlined in a previous paper [8], the optical transparency of glass fiber-reinforced composites depends on the following requirements: the fiber and polymer matrix should be either amorphous or their crystalline structures should be much smaller than the wavelength of visible light [9]. As E-glass fibers and an epoxy-based polymer matrix were used in this study, the amorphous material criterion applies. In the literature, both glass fibers and ribbons were used together with an epoxy-based polymer matrix [2,8,10,11,12,13,14,15,16,17,18,19,20,21]. On the other hand, nanofiber-reinforced transparent composites were produced using the above-mentioned principle that their structures are smaller than the wavelength of visible light [22,23,24,25,26,27,28,29,30,31,32].

The refractive indices (RIs) of the fiber and the polymer matrix must be as similar as possible in the wavelength range of visible light. Only in this case will the light neither become refracted nor scattered. However, the RI depends on the wavelength, and for every single material, according to its chemistry, the RI varies with wavelength, resulting in a dispersion curve. A rather simple quantitative indicator for the slope of the dispersion curve is the Abbe number. The higher a material’s Abbe number is, the less the RI changes over the visible wavelength spectrum and vice versa. In this regard, inorganic glass fibers have relatively high Abbe numbers compared to low-Abbe-number organic polymer matrices. To achieve the best possible match between fibers and the polymer matrix, the Abbe numbers of these materials should be as similar as possible. In case inorganic E-glass fabric and an organic epoxy matrix are used, their dispersion curves do not perfectly match and chromatic aberration occurs [12].

The infiltration quality is another critical requirement. Even if there is a perfect match between the RI of the polymer matrix and the fiber, low-quality infiltration will reduce the optical transparency due to voids. Voids are usually air pockets in or between yarns or textiles. As air has a substantially lower RI of ≈1.00 compared to that of the polymer matrix (≈1.55), air bubbles will act as refraction and scatter centers in the composite. Thus, an accurately low-porosity production system is crucial in transparent composite production.

The surface quality—surface smoothness—of the composite also has to be outstanding to achieve the best optical quality. Every surface defect, larger or comparable to the wavelength of visible light, will change the interface angle between the composite material and the surrounding medium. This changes the direction of the light passing through the composite and lowers the translucence.

The manufacturing of epoxy-based transparent fiber-reinforced composites is possible using simple experimental setups such as casting into a teflon-coated tray containing fibers [19], using hand-layup techniques [10,13] or pressing a fiber resin system stack between two glass plates using a vacuum bag for compaction [11]. Some other approaches feature vacuum-assisted resin transfer molding (VARTM) [2,14].

This research is a follow-up study to [8]. As concluded in this paper, there was a requirement to provide good surface quality with an RTM mold concept that is scalable for industrial applications, while providing sufficient process control to avoid porosity (voids). In this regard, a new mold was designed and produced. The same polymer system was applied together with a new mold design, which entails homogeneous E-glass fabric distribution in the composite cross-section, good process and flow front control, low porosity, and a smooth surface at the same time, resulting in good optical properties. In this article, the new design will be referred to as Optical-RTM. Based on this, the surface quality of the Optical-RTM- and standard RTM-produced samples were visualized and compared. This way, rather than an L-RTM setup requiring an artisanal, slow, and intensive labor force required, a more accurate and repeatable manufacturing method is achieved. Finally, its optical (dispersion curves of glass fibers and polymer as well as transmission over visible spectra curves of the tGFRP at varying tempering states), microstructural (fiber volume fraction and surface roughness), thermal (DSC and TGA), and mechanical (flexural) properties were evaluated, which helps in comparing the quality of the composites with those in the literature and also provides a direction for future research and development. The objective of this study was to identify the key factors for fabricating structural tGFRP specimens with high optical transmission using a new Optical-RTM mold design.

## 2. Materials and Methods

E-glass woven fabric and an epoxy resin system were used to produce tGFRP utilizing a new mold design and an RTM-based production technique (Optical-RTM). For validation, the RI of the polymer matrix and fibers were measured and three tGFRP plates were manufactured. Their properties were evaluated by measuring transmittance, glass transition temperature, onset and decomposition temperatures, fiber volume fraction, flexural strength and modulus as well as surface roughness.

### 2.1. Materials

The Materials selection has already been described in detail in a previous publication [8]. For this work, the same materials were chosen, with emphasis on the new production method. The materials are summarized in Table 1. For each material, different production batches were compared as well to check for consistency. The properties of the fabrics are given in Table 2 as well as are the data for the resin system in Table 3.

### 2.2. Polymer Synthesis

The protocol for synthesizing the polymer matrix was the same as that mentioned in a previous publication [8]. A 100:30 wt. ratio was used for epoxy resin L and hardener GL2.

### 2.3. Mold Design for Manufacturing tGFRP Plates (Optical-RTM)

**Figure 1:**
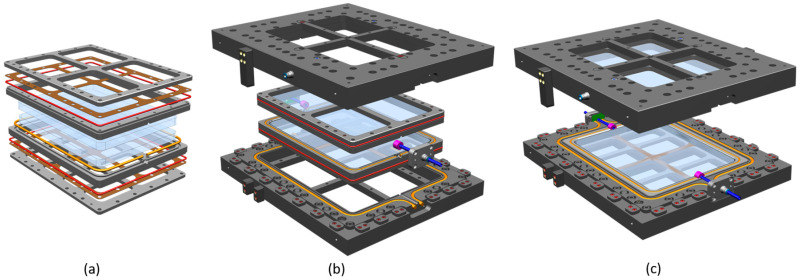
(**a**) Explosion view of glass plates and glass plate frame assembly, (**b**) explosion view of assembled glass plate frames and mold frames, and (**c**) assembled mold in open (charge) position.

**Figure 2:**
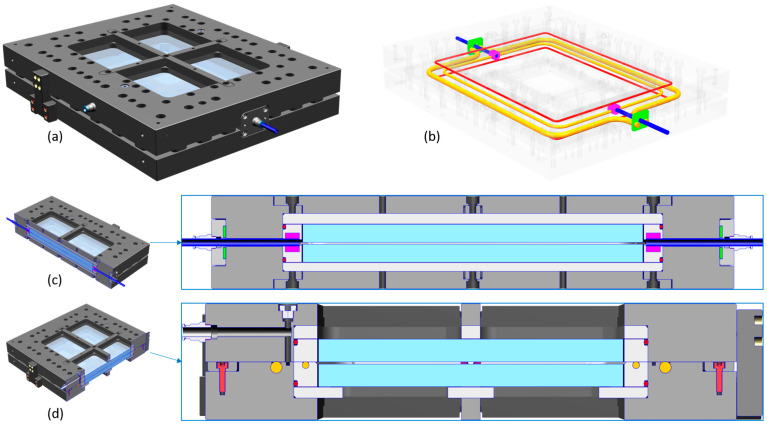
(**a**) Mold in closed (injection) position, (**b**) schematic illustration of the sealing concept, (**c**) cross-section through the mold’s middle in the flow direction, (**d**) cross-section perpendicular to the flow direction through the second evacuation line between the orange seals (top left).

The two main factors considered in the design of the Optical-RTM were a high surface quality and the minimization of voids through good process control during infiltration. Therefore, the tooling had to be suitably sealed to prevent air leakage into the cavity during evacuation to avoid porosity. A very low surface roughness, appropriate for the production of tGFRP, was provided by glass plates [8]. For these purposes, two glass plates (19 mm tempered safety glass of the type ESG/DIN EN 12150, [35] BE GLASS GmbH, Berlin, Germany) were glued with silicone rubber of the type Elastosil E43N (Wacker, Munich, Germany) into a metal frame with a circumferential glue gap of 1 mm (Figure 1a). The metal frames containing the glass plates were assembled with upper and lower metal plates, separated by 0.5 mm thick PTFE film (Figure 1a—brown: PTFE-Akzent GmbH, Bad Bramstedt, Germany). These assemblies were mounted in an upper and lower mold frame (Figure 1b), which contained centering devices and position stops to ensure a reproducible mold closing procedure and that the cavity height was in the closed position (Figure 2a). The fully assembled mold consisted of an upper and lower part, which connected at the parting plane when closed (Figure 1c and Figure 2a).

Sufficient vacuum and overpressure tightness was provided by a sealing system, consisting of three different levels (Figure 2b–d). The first level (red seals) ensured the vacuum tightness between the glass plate frames and the mold frames. Seals located in the mold parting plane (orange) prevented leakage during the evacuation of the air from the cavity as well as during pressure-injection of the resin system. The cylinder ring seal (magenta) and the vertical shutoff plate seal (green) formed the third level and closed the parting plane seal loops (orange) while allowing the injection and evacuation line (blue) to reach the cavity. The red (4 mm Ø) and orange (10 mm outer Ø and 6 mm inner Ø) round cord seals were made from GP60T-type silicone (MVQ Silicones GmbH, Weinheim, Germany) with a 60 Shore A hardness, and the green vertical shutoff seal was cut from a 3 mm thick GP40T-type silicon plate (MVQ Silicones GmbH, Weinheim, Germany) with a 40 Shore A hardness, while the magenta cylinder ring seal was cast from KDSV M 4470 silicone and Catalyst 40 (R&G Faserverbundwerkstoffe GmbH, Waldenbuch, Germany). To prevent leakage into the cavity, a second evacuation line was connected to the volume in between the two orange seals (Figure 2d). Given that the evacuation line of the cavity had a higher absolute air pressure than the evacuation line between the two orange seals did, any leakage would have occurred from the cavity towards the evacuated volume between the orange seals preventing any potential leakage from entering the mold cavity.

### 2.4. Manufacturing of tGFRPs

The Optical-RTM mold was used to manufacture high-quality tGFRP samples. Prior to manufacturing, the glass surfaces of both mold parts were pretreated with the release agent Marbocote HP7 (Marbocote Limited, Middlewich, UK) after cleaning with isopropanol. A total of three coats were applied, with a flash-off time of 10 min between the coats. The glass fiber preform was made from two 14-layer woven fabric stacks, which were sewn together, resulting in a 28-layer final preform. The dimensions of the preform matched the cavity dimensions of 360 mm × 260 mm. Since the RI of the polyester sewing yarns was different from the RI of the epoxy resin system, the sewing yarns were only used at the edges to secure the fabric plies of the preform during handling. The sewing areas were excluded from mechanical, optical, and thermal testing procedures. After a careful insertion of the preform into the mold cavity, possible runners at the edges of the preform were sealed with tacky tape (SLT150B, Composyst GmbH, Landsberg am Lech, Germany (Figure S1a, Supplementary Material)) and the mold was closed and secured with 26 M12 cylindrical head screws and a torque wrench with a maximum torque of 50 Nm. The inlet hose was connected to the pressure pot, which held a metal can containing the pre-mixed epoxy resin system, while the outlet was connected to a vacuum pump. Additionally, the in-between seal evacuation was connected to an additional vacuum pump set to 60 mbar. During the RTM process, the resin was transported into the mold by the pressure difference created by the vacuum pump at the outlet and the pressure level at the inlet. The flow front was controlled by manually adjusting the inlet and outlet pressures during injection, resulting in a constant flow rate. During the infiltration, initially, no pressure was applied on the injection line while the absolute pressure on the evacuation line was slowly reduced from ambient pressure (≈965 mbar) to 160 mbar. After reaching 160 mbar on the evacuation line, the velocity of the flow front was controlled by applying pressure on the injection side. A maximum pressure of 2 bar was not exceeded. After the mold was filled, the injection and evacuation lines were clamped and the resin system was cured at room temperature for 24 h.

**Figure 3:**
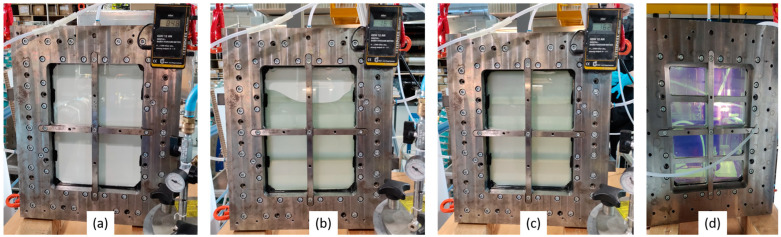
(**a**) Start of the infusion by evacuation of the mold cavity, (**b**) flow front propagation, (**c**) filled mold, and (**d**) tGFRP in the cavity after 24 h curing at room temperature.

Figure 3 shows the flow front propagation and curing process in further detail. The absolute gas pressure in the cavity was slowly reduced until the liquid resin system reached the fibers on the lower side of the mold (a). The mold design allowed an almost linear flow front during the injection process (b). In addition, there was a complete filling of the cavity and avoiding collisions with the flow front that would have led to the entrapment of residual air in the collision area (Figure S2, Supplementary Material) was no longer a challenge. The appearance of potential runners was prevented with tacky tape and by the ability to apply additional controlled pressure on the injection side while the flow front could be visually inspected (b,c). After 24 h of curing at room temperature, the RI of the polymer matrix was already much closer to the fibers’ RI (d) compared to that of the liquid resin system (c). All samples were stuck to the upper mold side when opened (Figure S1b, Supplementary Material). Mechanically removing them would have risked damaging the mold’s glass cavity. Hence, they were tempered, while they were attached to the upper mold for 15 h at 60 °C. After tempering, the tGFRP plates could be easily removed from the upper mold without the use of tools. A total of three plates were produced and samples were taken from each plate for the following analyses.

### 2.5. Optical Evaluation

#### 2.5.1. Refractive Index Measurements

The RIs of the pure polymer and the woven E-glass fabric were measured to evaluate the mismatch. The RI of the polymer was measured over the visible spectrum (350–800 nm) with a Woollam RC-UI ellipsometer (J.A. Woollam Co., Lincoln, NE, USA). The RI dispersion curve of the glass fiber was measured using an indirect method comparable to the wavelength scan method reported by Kang et al. [36]. For this indirect measurement, three layers of glass fabric were embedded in RI liquids from Cargille Laboratories (Cedar Grove, NJ, USA) with known dispersion curves. The transmittance of those samples was measured with a compact CCD spectrometer from Thorlabs CCS 200 (Thorlabs GmbH, Bergkirchen, Germany). The wavelength, at which the maximum transmittance value was obtained in a particular standard liquid and fabric combination, was determined from the dispersion curve of that standard liquid to identify the exact RI value of that liquid at that wavelength. By repeating that process with a series of different standard liquids, data points for the dispersion curve, i.e., RI vs. wavelength, of the glass fabric were obtained. The glass fiber dispersion curve was estimated with the Cauchy equation (Equation (1)) by using the three constants (A, B, and C). In this case, A = 1.5425, B = 6.754 × 10^3^, and C = −3.585 × 10^8^.

(1) $$n\left(\lambda \right)=A+\frac{B}{\lambda^{2}}+\frac{C}{\lambda^{4}}$$

#### 2.5.2. Transmittance Measurements

Transmittance was measured with a compact Thorlabs CCS 200 CCD spectrometer (Thorlabs GmbH, Bergkirchen, Germany) using a Thorlabs QTH10/M halogen lamp with a broad emission spectrum from 400–2200 nm (Figure S3, Supplementary Material). The light was focused with a lens (F = 35 mm) and an aperture on the sample, which was placed in a sample holder. The transmission was calculated by dividing the transmission intensity of an empty reference measurement of the light source by the measured intensity with the specimen inserted in the light path. From each plate, three (25 × 25 mm^2^) samples were cut and measured at three different points.

### 2.6. Microstructural Evaluation

#### 2.6.1. Microscopic Analysis

Optical reflected light microscopy (ZEISS Axioskop with differential interference contrast) and an analysis via a scanning electron microscope (ZEISS Auriga field-emission SEM) were used for the evaluation of the tGFRP cross-section for each plate. Specimens were taken from the center of the plates and were prepared in accordance with the method of the previous publication [8].

#### 2.6.2. Surface Roughness Analysis

The surface roughness of the composites produced by the standard RTM and Optical-RTM molds were compared using white light interferometer measurements on Zygo Nexview™ NX2 (Ametek Inc., Berwyn, PA, USA). A 5.5× Michelson microscope with an objective lens with a 0.15 numerical aperture was used. The visualized area for each sample was ≈9.88 mm^2^ (≈3144 µm on each axis).

#### 2.6.3. Fiber Volume Fraction

The average global fiber volume fraction ($\varphi_{g}$, Equation (2)) for each plate was calculated from the thickness (h) measurements of the four-point bending specimens and 28 textile layers (n) with an aerial weight ($M_{F}$) of 106 g/m^2^ as well as an E-glass fiber density ($\rho_{F}$) of 2.59 g/cm^3^ [37].

(2) $$\varphi_{g}=\frac{M_{F}\times n}{\rho_{F}}\times \frac{1}{h}\times 100\%$$

Additionally, the local fiber volume fraction of the micrograph cross-sections ($\varphi_{l}$, Equation (3)) was evaluated as well as was the fiber volume fraction of individual yarns, also known as yarn packing density ($p_{d}$, Equation (4)). For this assessment, SEM images were first converted into 8-bit grayscale images and further transformed into black and white (BW) images with adjusted threshold values for separating the polymer matrix from the fibers using the open-source software ImageJ version 1.54b [38]. A mask of the BW image was then generated with the ‘analyze particle’ function deleting every particle smaller than 5 pixels to remove artifacts resulting from lighting during the recording of the micrograph images (Figure S5, Supplementary Material).

A section of the BW image mask was used to analyze the local fiber volume fraction from the number of black (fibers, $n_{F}^{\mathrm{Pixel}})$ and white (matrix, $n_{M}^{\mathrm{Pixel}})$ pixels within a selected area.

(3) $$\varphi_{l}=\frac{n_{F}^{\mathrm{Pixel}}}{n_{F}^{\mathrm{Pixel}}+n_{M}^{\mathrm{Pixel}}}\times 100\%$$

Furthermore, the BW image masks were used to find the number ($n_{F})$ and diameter ($D_{F})$ of all fibers within a yarn cross-section and calculate the yarn packing density. The fibers (coordinates of the center points and radii) were detected using Matlab R2022a with the imfindcircles function [39], while the convex hull (x and y points of the smallest convex area including the sum of all points on the fiber diameters) of the yarn was determined with the convhull function [40]. The area of the convex hull ($A_{y})$ was calculated by the polyarea function [41].

(4) $$p_{d}=\frac{1}{A_{y}}\;\overset{n_{F}}{\underset{i=1}{\sum }}\frac{\pi }{4}D_{\mathrm{Fi}}^{2}\times 100\%$$

### 2.7. Thermal Evaluation

#### 2.7.1. Thermogravimetric Analysis (TGA)

TGA was performed on TGA 2950 (TA Instruments, New Castle, DE, USA) under N_2_ atmosphere starting from 25 °C to 900 °C with a heating rate of 10 °C/min.

#### 2.7.2. Differential Scanning Calorimetry (DSC)

DSC analysis was conducted for each plate at three different curing states (the 1st was for 24 h at RT + 15 h at 60 °C, the 2nd was for an additional 24 h at 60 °C, and the 3rd was for an additional 48 h at 60 °C). After each curing state, a DSC sample was taken from each plate and the plates were further cured. DSC 2920 (TA Instruments, New Castle, DE, USA) was operated under N_2_ atmosphere starting from 25 °C to 150 °C with a heating rate of 10 °C/min.

### 2.8. Mechanical Evaluation

#### Characterization of Flexural Properties

Four-point bending tests were carried out to evaluate the mechanical properties in accordance with the DIN EN ISO 14125 [42] standard using an Inspekt 20 kN testing machine (Hegewald & Peschke Meß- und Prüftechnik GmbH, Am Gründchen, Germany). The testing apparatus is shown in Figure S4 (Supplementary Material).

The testing parameters used depended on the sample thickness and are listed in Table 4. The specimen length (l) was 85 mm, the radii of the support and pressure fin were 2 mm and the testing speed was 5 mm/min for all samples tested. Six specimens per plate were tested. The thickness of the sample was measured with an outside micrometer and the width was measured with a standard caliper gauge.

## 3. Results

### 3.1. Optical Properties

#### 3.1.1. Adjusting the Refractive Index

**Figure 4:**
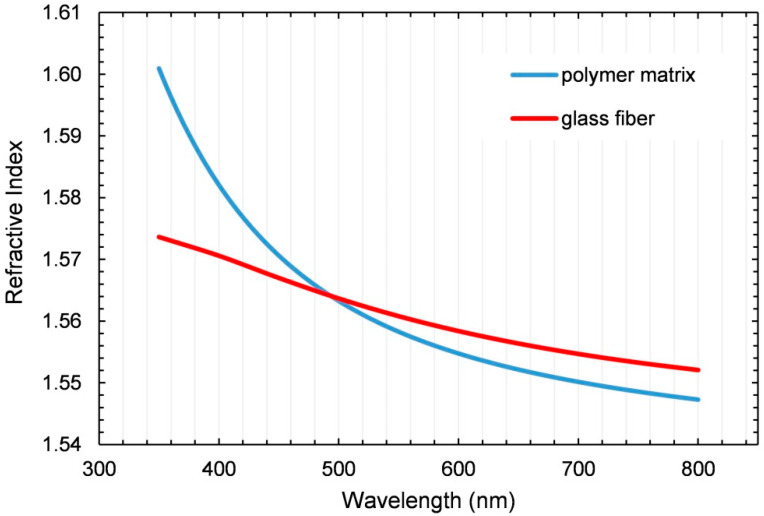
Polymer and glass fiber dispersion. Reproduced with permission from [43].

The dispersion curves of the glass fabric and the cured polymer matrix are shown in Figure 4. The intersection point was at λ = 494 nm. The absolute mismatch for five different wavelengths is presented in Table 5. The stronger increase at shorter wavelengths for the polymer led to a pronounced chromatic aberration (Δn_400nm_ = 0.0117).

The tempering process increased the density and thereby the RI of the polymer. Thus, the RI at a wavelength of λ = 589 nm increased from 1.5393 to 1.5555 (Δn = 0.0162) during the tempering process.

**Figure 5:**
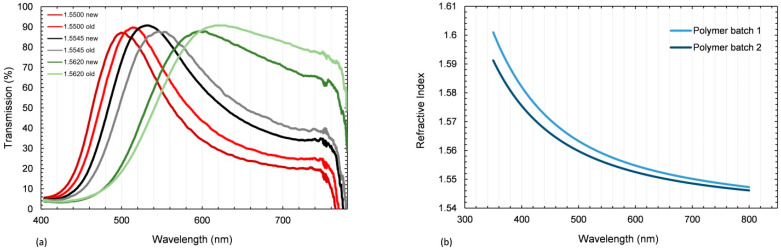
(**a**) Transmission over wavelength curves for different batches with three layers of E-glass fabric immersed in different RI liquids, and (**b**) dispersion curves of the pure cured polymer from different batches.

To ensure the reproducibility of the tGFRPs manufactured from commercially available products of different material batches (fiber and resin), their RIs were analyzed and compared in Figure 5. This comparison revealed a significant variation in the RI of both the glass fibers and epoxy resin system from different batches. For the glass fiber, the transmission shift was analyzed. For these purposes, three layers of fabric were immersed in three different standard RI liquids. The comparison showed that the peak of transmission shifted to the left for the new fiber batch. This can be explained by the slightly higher refractive index of the new fiber batch. As the RI of the glass fiber is dependent on the glass density [44] it can easily change with different glass compositions [14] or other factors such as cooling speed [44] and filament diameter [36] during fiber production. The epoxy resin system also showed a change in the RI over the visible wavelength spectrum for different batches of the material, as analyzed by an ellipsometer measurement. The change in ${\Delta n}_{D}^{20}$ was 0.0024 and can be attributed to slight changes in resin composition and the resin–hardener ratio [14].

#### 3.1.2. Transmission Results

**Figure 6:**
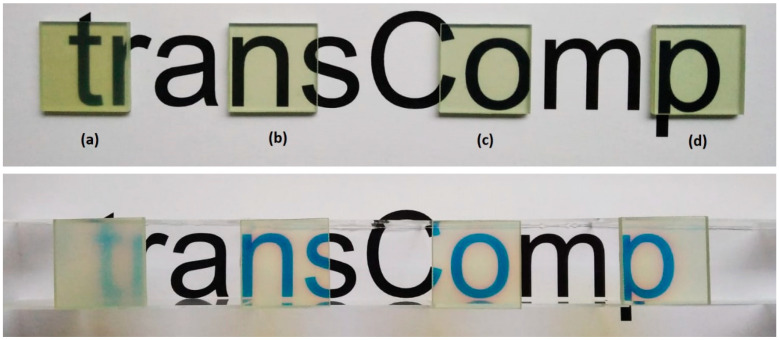
Photographs of (**a**) standard and (**b**–**d**) Optical-RTM samples: (**b**) 87 h, (**c**) 39 h, and (**d**) 15 h tempering at 60 °C; (upper row) sample directly on the text and (lower row) sample 2.5 cm above.

**Figure 7:**
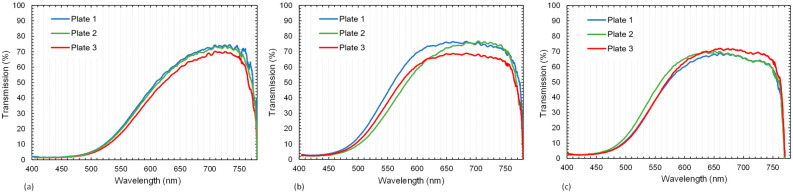
Transmission results for the tGFRP plates 1–3: (**a**) after 24 h at room temperature with curing for 15 h at 60 °C, (**b**) additional curing for 24 h at 60 °C and (**c**) additional curing for 48 h at 60 °C.

Photographs of the composites are shown in Figure 6. On the left side (a) is a standard RTM-produced sample from a previous publication [8] with a rather high surface roughness. The effect of surface roughness is especially visible in the lower row, where the samples were positioned 2.5 cm above the paper. The samples from (b) to (d), which were all produced by the Optical-RTM mold, differed from each other in their tempering time. Both in the upper and lower rows, no practical difference can be detected by naked-eye observation. They all show chromatic aberration in the lower row images. However, the transmittance, as analyzed in Figure 7, shows differences.

**Figure 8:**
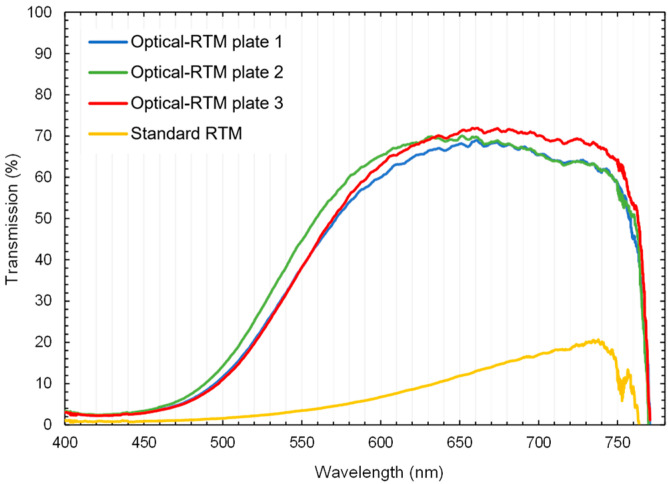
Comparison of standard and Optical-RTM transmission over wavelength curves for tempering state lasting 24 h at RT + 87 h at 60 °C.

Figure 7a represents the results of the transmission measurement for three different plates cured for 15 h at 60 °C. Curves were smoothened using a moving average of over 20 data points. All samples consisted of 28 layers of E-glass fabric and epoxy polymer. They show comparable curves with a maximum transmission of around 74% at 715 nm. For samples that were further tempered at 60 °C for 24 h, the peak region of the transmission curve shifted towards shorter wavelengths (Figure 7b). Additional tempering for 48 h at 60 °C did not lead to a significant change in comparison to the 24 h curing cycle. This indicates that further tempering has no significant effect on transmission, although the T_g_ of the polymer increased (Chapter 3.3.2). Additionally, Figure 8 provides a comparison of the transmission curves between a standard RTM sample from the previous work [8] and the tempered Optical-RTM samples from Figure 7. The influence of surface quality is significant. The Optical-RTM led to higher transmission due to the use of a glass surface instead of an aluminum and poly(methyl methacrylate) (PMMA) tool, as used before [8].

### 3.2. Microstructural Properties

#### 3.2.1. Microscopic Analysis

**Figure 9:**
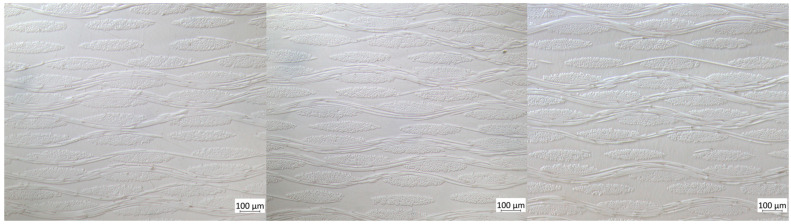
Optical microscopy images of tGFRP plate 1 (**left**), 2 (**middle**), 3 (**right**).

**Figure 10:**
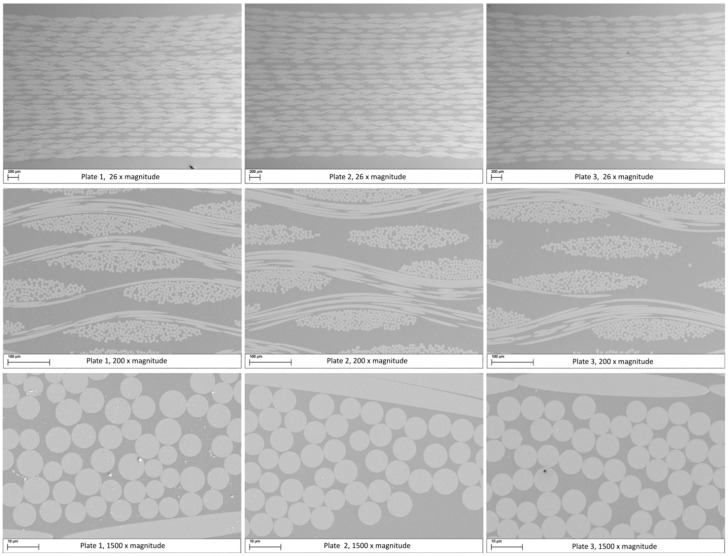
SEM micrographs of plates 1, 2, and 3 (left to right) at 26×, 200×, and 1500× magnification (top to bottom).

The infiltration quality was evaluated from optical microscopy (Figure 9) and SEM analysis (Figure 10). The fabrics were homogeneously distributed throughout each sample. The microscopic interfibrillar spaces inside the yarns, as well as the macroscopic spaces between the yarns and fabrics, were fully impregnated and showed no visible porosity.

#### 3.2.2. Surface Roughness

**Figure 11:**
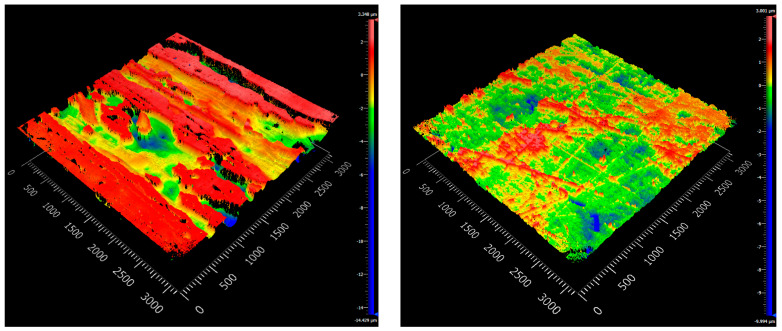
White light interferometer micrographs of tGFRPs produced by standard RTM mold (**left**: top; **right**: bottom surfaces).

**Figure 12:**
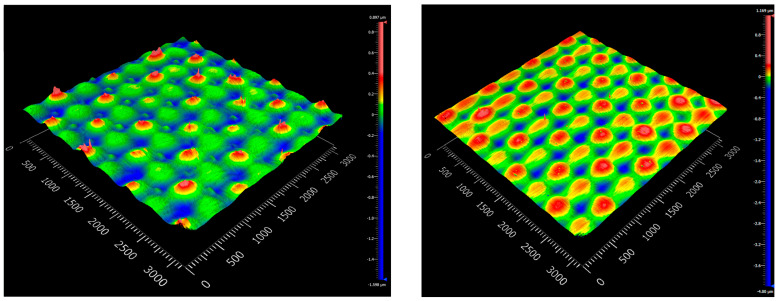
White light interferometer micrographs of tGFRP produced by Optical-RTM mold (**left**: top; **right**: bottom surfaces).

The surface roughness of the composites was visualized and evaluated by white light interferometry. The top and bottom topographical images of the samples produced with the standard and Optical-RTM molds are shown in Figure 11 and Figure 12. Graphics presenting local deviations in the topography of the surfaces are shown in Figures S10–S15 (Supplementary Material). A distinct difference between the surface qualities of the samples produced with the standard and Optical-RTM molds can be observed. While the top surface of the standard RTM mold was made of poly(methyl methacrylate) (PMMA), the bottom layer was aluminum, resulting in poor surface quality. By contrast, the only surface feature of the Optical-RTM molds on both the top and bottom surfaces was the pattern of the woven E-glass fabric. Other than that, no surface feature was visible due to the glass cover material of the Optical-RTM mold. A comparison of the surface roughness parameters Sa and Sq, which are the arithmetic average of the profile height deviation, and the root mean square average of profile height deviation, respectively, are listed in Table 6 for four different surfaces of the compared samples. These results indicate that the surfaces produced by Optical-RTM had a reduced surface roughness of more than 15 times that of the surfaces prepared by standard RTM. These findings match the results for the transmission comparison for both mold designs presented in Figure 8.

#### 3.2.3. Fiber Volume Fraction Results

**Figure 13:**
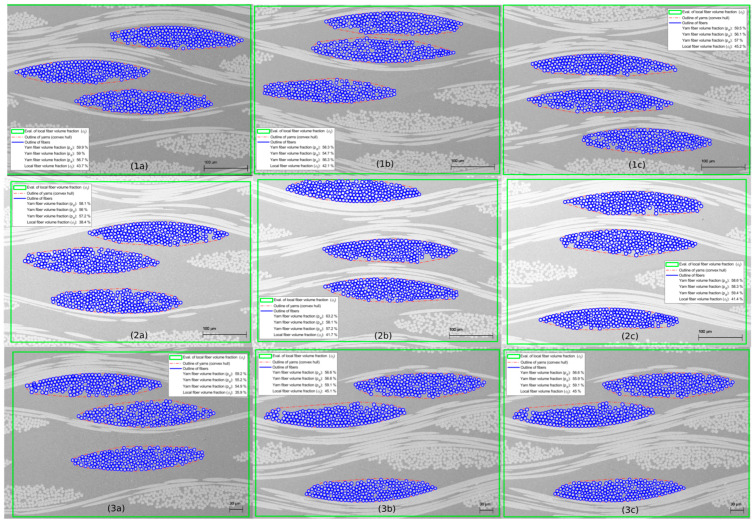
Yarn ($p_{d}$) and local fiber volume fraction ($\varphi_{l}$) analysis results of plates 1–3 at three different cross-section locations (**a**–**c**).

**Figure 14:**
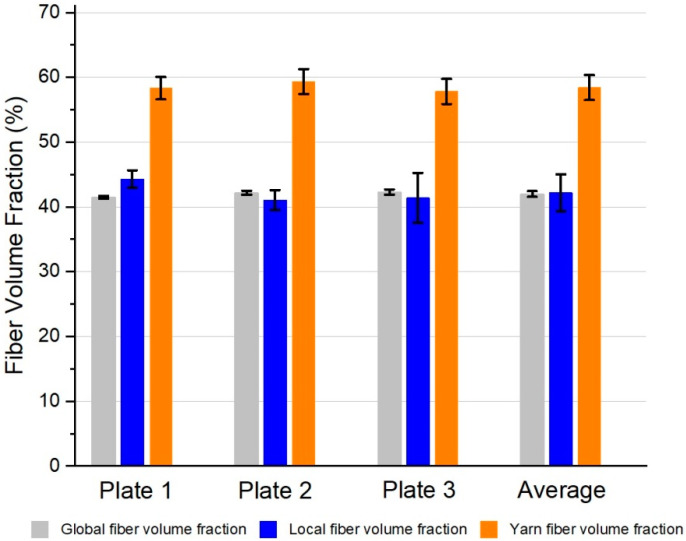
Comparison of global, local, and yarn fiber volume fraction results.

The results for the global fiber volume fraction ($\varphi_{g}$) are presented in Table 7. The mean fiber volume fraction among all specimens of all plates was 42% with a standard deviation of 0.5%. The results of the local ($\varphi_{l}$) and yarn ($p_{d}$) fiber volume fraction are shown in Figure 13 and Table 8. The mean local fiber volume fraction for all specimens was 41.6% with a standard deviation of 2.8%, while the average yarn package density was 57.6% with a standard deviation of 1.9%. All results are summarized in Figure 14.

### 3.3. Thermal Properties

#### 3.3.1. Thermogravimetric Analysis Results

**Figure 15:**
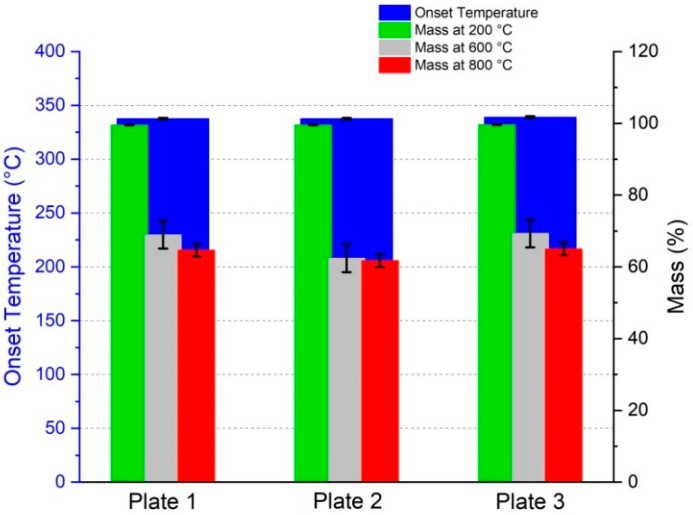
TGA results: Onset temperature as well as a mass loss at 200 °C, 600 °C, and 800 °C.

The TGA results are summarized in Figure 15 as well as Table 9; the TGA curves are shown in Figure S6 (Supplementary Material).

#### 3.3.2. Differential Scanning Calorimetry Results

**Figure 16:**
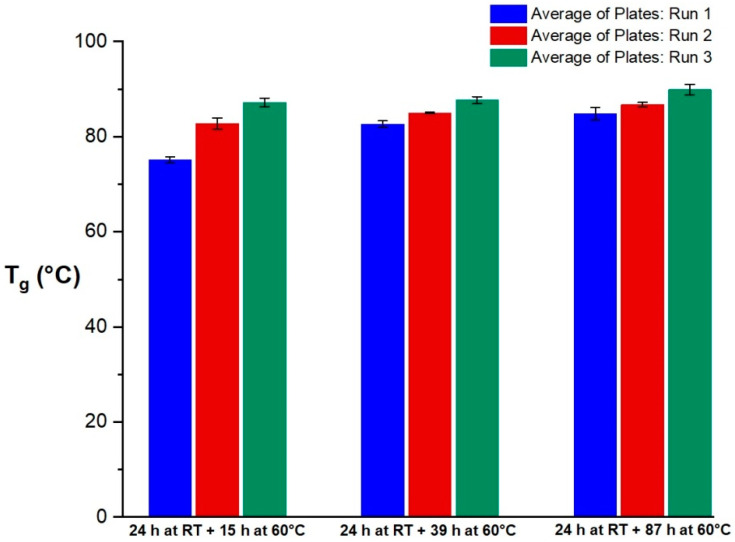
Average T_g_ values determined by DSC of three tGFRP plates for three DSC runs and three curing states.

The DSC results of the tGFRP specimens of different tempering cycles are shown in Figure 16 as well as Table 10; the corresponding DSC data are summarized in the Supplementary Material (Figures S7–S9 and Tables S1–S3).

### 3.4. Mechanical Properties

#### Flexural Properties

**Figure 17:**
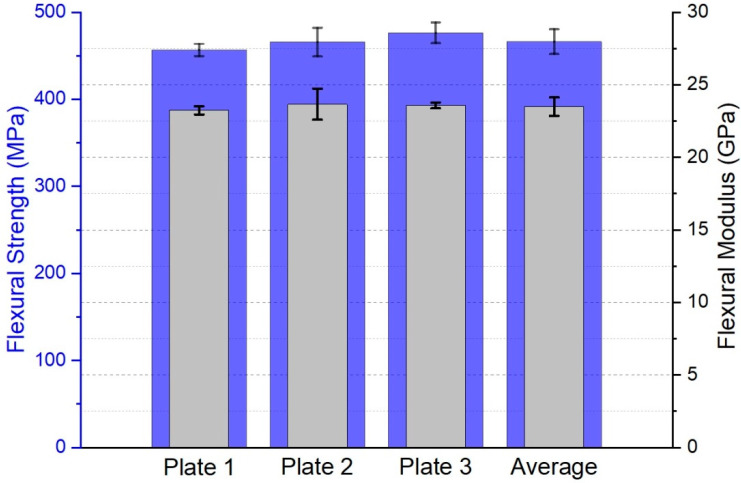
Four-point bending results (flexural strength (blue) and modulus (gray)).

The results of the four-point bending characterization are presented in Table 11 and Figure 17. The force–displacement curves are shown in Figure S16 (Supplementary Material).

The datasheet of the epoxy resin system [34] provides mechanical data for a comparison with the experimental results. A flexural strength of 431 MPa was achieved for a non-transparent glass fiber-reinforced polymer (GFRP) manufactured by hand lamination. The GFRP datasheet consists of the same resin and hardener (L + GL2) in combination with 12 layers of 296 g/m^2^ satin-woven E-glass fabric. The GFRP was cured using the same tempering cycle and was tested in accordance with the DIN EN ISO 14125 [42] three-point bending standard. The GFRP had a thickness of 3 mm and a theoretical fiber volume fraction of 45.7% (Equation (2)).

## 4. Discussion

In this study, a new mold concept was realized to meet the requirements of tGFRP composite production with the RTM technique. The major requirement to produce such a mold (Optical-RTM) was to provide sufficient smoothness to the cavity’s surface. With the topological analysis of the tGFRP surface, the main advantage of the Optical-RTM mold technique outlined here was visualized. Due to its glass cavity, no additional surface roughness was created on either side of the composite. The only noteworthy aspect of those surfaces was that they displayed the pattern of the E-glass fabric. The reason for its appearance can be attributed to the shrinkage of the polymer matrix during the polymerization process. In our previous publication [8], samples with 29 layers of woven E-glass fabric provided a maximum transmission of 75%; here, 74% was achieved. Due to the difference between the dispersion curves of the polymer matrix and fiber, there is no simple correlation between the refractive index and transmission.

The Optical-RTM mold also provided good process control due to the possibility to manipulate the pressure difference between the inlet and outlet of the cavity while visually inspecting the flow front. The infiltration of a polymer matrix into the interfibrillar space is the most prominent factor for the production of a composite to be considered successful. The optical light and scanning electron microscopy images prove that the the advanced level of infiltration quality was achieved without visible voids and defects.

In addition to surface quality and void content, a sufficient match between the RIs for the fully cured polymer and the E-glass fiber was required to manufacture a high-quality tGFRP sample. Measuring the dispersion curves of the polymer and the glass fiber showed that the overall slope of the polymer’s dispersion curve was steeper than the slope of the glass fiber’s curve. This phenomenon was also described in an earlier publication [12]. It becomes clear that the RI mismatch depends on the wavelength, with a chromatic aberration [12] occurring for those wavelengths at which the RIs differ more. Furthermore, a major influence on the polymer’s RI is the tempering cycle.

The transmission measurements showed consistent results for the three different plates at each curing state. The peak of the transmission curves shifted with the increasing tempering time from higher to lower wavelengths. After the second cycle which ran for an extra 24 h at 60 °C, there was no visible change in the transmission curves of the composites compared to the results of the third cycle running for an extra 48 h at 60 °C. The transmission curves of these three different tempering states correlate with the DSC results. Thus, the second and third tempering states showed similar glass transition temperatures while there was a clear increase in T_g_ from the first tempering to the second. The TGA results showed consistent properties for all three tGFRP plates. Furthermore, all plates had a glass fiber content of approximately 63 wt.-%. The average global fiber volume fraction of all three plates was between 41.9 and 42.7 v.-%, which is in good agreement with the local fiber volume fraction of 41.6 ± 2.8 v.-%. As expected for FRPs in general, the yarn packaging density was higher at 57.6 ± 1.9 v.-%.

The mechanical properties obtained in this study are very consistent for all of the three tested plates, which is plausible because most of the mechanical properties of composite materials are mainly dependent on the fiber content, which was also very consistent for all samples. The results showed a slightly higher flexural strength (466.29 ± 14.29 MPa with a global fiber volume fraction of 42 ± 0.5%) in comparison to standard non-transparent GFRP properties manufactured by hand lamination of the same epoxy resin system but a different type of woven E-glass fabric (431 MPa with a global fiber volume fraction of 45.7%) [34]. This may be a result of the good surface quality (very low surface roughness) and low void content (not detectable via microscopy methods) of the specimen produced by the Optical-RTM setup.

The consistency of the fiber volume fractions, infiltration quality, and flexural and optical properties across the three plates manufactured indicate that the Optical-RTM mold design, in combination with the RTM production technique presented, is suitable for producing tGFRPs with reproducible properties.

On the other hand, there is a significant difference between the transmittance vs. wavelength curves of the L-RTM-derived sample of our previous study [8] and those of the current RTM-produced samples, although the same epoxy system and the same fiber and fabric type were utilized. To investigate the source of that difference systematically, the measurements outlined in Figure 5 were carried out. The results prove that there are RI discrepancies in both the fibers and the polymer matrix in different batches. In addition, possible deviations in the degree of polymer curing, which take place during the polymerizations in L-RTM and RTM techniques, can be made accountable. As is known, during L-RTM production, the monomer mixture infiltrate the mold by the vacuum, while in RTM, the same happens with additional pressure. This major principal difference in the techniques might be responsible for the differences in reactivity, density, and eventually the RI of the composite. As the focus of this study was the development and performance of the Optical-RTM mold, future research will investigate this phenomenon in a detailed manner.

## 5. Conclusions

The Optical-RTM mold served well in its design and production purposes. Surface quality, which is one of the major parameters for the optical transmission of a material, was varied in a controlled manner using the same chemistry, fiber, and textile structures. The degree of its effect was both visualized and quantified. The infiltration quality of the product was visually proven to be outstanding. In this work, the production of tGFRPs with approximately 74% maximum transmittance, a fiber volume fraction of ≈42% and a sample thickness of 2.7 mm was achieved; nevertheless, chromatic aberration still existed due to a dispersion curve mismatch between the fiber and polymer matrix. Different commercially available batches of fabrics and resin systems may differ in RI properties. This requires a measurement of the fabric and fully cured polymer RIs for each new material batch. Overall the most important factors for tGFRP production have been identified:

- High surface quality of the production mold.
- No detectable void content being ensured through a suitable production method and flow front control.
- Refractive index matching of the fiber and polymer over majority of the visible spectrum.

## Figures and Tables

**Table 1:** List of materials forming the tGFRP.

Type	Material
Fiber (Fabric)	E-Glass Fabric HexForce 02116 1260 TF970 ^1^
Sewing yarn	Serafil 180 Tex 16 ^2^
Epoxy Resin	Epoxy Resin L ^3^
Hardener	Hardener GL2 ^3^

^1^ Wela Handelsgesellschaft mbH, Geesthacht, Germany. ^2^ Amann&Söhne GmbH&Co. KG, Bönnigheim, Germany. ^3^ R & G Faserverbundwerkstoffe GmbH, Waldenbuch, Germany.

**Table 2:** Material properties of the glass fabric [33].

Properties	E-Glass Fabric HexForce 02116 1260 TF970
Type of weave	Plain woven
Type of yarn	EC7 22 glass
Type of finish	Silan-based type TF970
Yarn/weight distribution warp	24 yarn/cm/51%
Yarn/weight distribution weft	23 yarn/cm/49%

**Table 3:** Material properties of epoxy resin L with hardener GL2 [34].

Properties	Epoxy Resin L and Hardener GL2
Mixing ratio by weight	100:30
Gel time of 100 g at 20 °C	210 min
Mixed viscosity	248 mPa s
Flexural strength polymer	119 MPa

**Table 4:** Parameters for four-point bending testing.

Parameter	Plate 1	Plate 2	Plate 3
Average thickness, h (mm)	2.769	2.715	2.711
Average width, w (mm)	14.89	14.91	14.92
Support distance, L (mm)	64	62	62
Pressure fin span, L′ (mm)	21	21	21

**Table 5:** Refractive index mismatch of fiber and polymer.

Wavelength (nm)	Δn ^1^
400	0.0117
500	0.0004
600	0.0036
700	0.0045
800	0.0048

^1^ Refractive index mismatch at a given wavelength between glass fiber and epoxy resin system.

**Table 6:** Surface roughness parameters for the top and bottom sample surfaces.

Surface Roughness	Standard RTM Top	Standard RTM Bottom	Optical-RTM Top	Optical-RTM Bottom
**Sa (µm)**	1.535	0.618	0.078	0.090
**Sq (µm)**	1.897	0.780	0.106	0.113

**Table 7:** Global fiber volume fraction results (calculated from bending specimen thickness).

Specimen	Plate 1 Thickness (mm)	Plate 1 $\varphi_{g}$ (%)	Plate 2 Thickness (mm)	Plate 2 $\varphi_{g}$ (%)	Plate 3 Thickness (mm)	Plate 3 $\varphi_{g}$ (%)
1	2.772	41.3	2.702	42.4	2.682	42.7
2	2.781	41.2	2.692	42.6	2.689	42.6
3	2.788	41.1	2.695	42.5	2.701	42.4
4	2.748	41.7	2.741	41.8	2.732	41.9
5	2.763	41.5	2.732	41.9	2.727	42.0
6	2.763	41.5	2.729	42.0	2.736	41.9
Average	2.769	41.5	2.715	42.2	2.711	42.3
Standard Deviation	0.015	0.2	0.021	0.3	0.023	0.4

**Table 8:** Average and standard deviation (SD) of local ($\varphi_{l}$) and yarn ($p_{d}$) fiber volume fractions.

Plate	Section	$\varphi_{l}$ [%]	Average $\varphi_{l}$ [%]	SD $\varphi_{l}$ [%]	$p_{dl}$ [%]	$p_{d2}$ [%]	$p_{d3}$ [%]	Average $p_{d}$ [%]	SD $p_{d}$ [%]
1	a	43.7	43.7	1.3	59.9	59.0	56.7	57.5	1.7
	b	42.1			58.3	54.7	56.3		
	c	45.2			59.5	56.1	57.0		
2	a	38.4	40.5	1.5	58.1	56.0	57.2	58.5	1.9
	b	41.7			63.2	58.1	57.2		
	c	41.4			58.6	58.3	59.4		
3	a	35.9	40.8	3.8	59.2	55.2	54.9	57.0	1.9
	b	45.1			56.6	56.6	59.1		
	c	41.3			60.1	54.8	56.4		
Average			41.6	2.8				57.6	1.9

**Table 9:** TGA results.

tGFRP	Onset Temperature [°C]	Decomposition Temperature [°C]	Mass at 200 °C [%]	Mass at 600 °C [%]	Mass at 800 °C [%]
Plate 1	337.67	354.94	99.54	68.99	64.65
Plate 2	337.70	351.60	99.55	62.42	61.75
Plate 3	339.12	354.00	99.60	69.36	65.05
Average	338.16	353.51	99.56	66.92	63.82
Standard deviation	0.83	1.72	0.03	3.90	1.80

**Table 10:** Average T_g_ values determined by DSC over three tGFRP plates for three DSC runs and three curing states.

Curing State	Average T_g_ DSC Run 1 [°C]	Standard Deviation DSC Run 1 [°C]	Average T_g_ DSC Run 2 [°C]	Standard Deviation DSC Run 2 [°C]	Average T_g_ DSC Run 3 [°C]	Standard Deviation DSC Run 3 [°C]
24 h at RT + 15 h at 60 °C	75.2	0.6	82.7	0.6	84.8	1.3
24 h at RT + 39 h at 60 °C	82.8	1.2	85.0	0.2	86.7	0.5
24 h at RT + 87 h at 60 °C	87.2	0.9	87.7	0.8	89.9	1.1

**Table 11:** Flexural properties.

tGFRP	Average Flexural Strength (MPa)	Standard Deviation Flexural Strength (MPa)	Average Flexural Modulus (GPa)	Standard Deviation Flexural Modulus (GPa)
Plate 1	456.73	7.00	23.24	0.28
Plate 2	465.75	16.46	23.66	1.07
Plate 3	476.38	11.92	23.58	0.20
Average	466.29	14.29	23.49	0.64

## Data Availability

Data Sharing is not applicable.

## Supplementary Materials

The following supporting information can be downloaded at: https://www.mdpi.com/article/10.3390/polym15092183/s1. Figure S1: (a) Preform in the mold cavity and tacky tape (black) applied to prevent runners on the edges, (b) tGFRP plate sticking to the upper mold when opening after 24 h of curing at room temperature; Figure S2: (a) Preform with cutting tolerances placed in a stiff mold with defined cavity dimensions leading to a fiber-free volume between the preform and mold, (b) race-tracking (runners) on the preform edges, and (c) uncontrolled flow fronts colliding; Figure S3: Spectrometer for transmittance measurements; Figure S4: Four-point bending apparatus (left: backside, right: front side); Figure S5: Image preprocessing using ImageJ— (a) 8-bit grayscale, (b) BW image with threshold values, and (c) mask of BW image; Figure S6: TGA results of plates (a) 1, (b) 2 and (c) 3; Figure S7: DSC results of tGFRP specimens after curing for 24 h at room temperature with tempering for 15 h at 60 °C; Figure S8: DSC results of tGFRP specimens after curing for 24 h at room temperature and 39 h tempering at 60 °C; Figure S9: DSC results of tGFRP specimens after curing for 24 h at room temperature and 87 h tempering at 60 °C; Figure S10: White light interferometer data of the top surface of the Optical-RTM-derived sample; Figure S11: White light interferometer data of the bottom surface of the Optical-RTM-derived sample; Figure S12: White light interferometer data of the top surface of the standard RTM-derived sample; Figure S13: White light interferometer data of the bottom surface of the standard RTM-derived sample; Figure S14: 2D view of the white light interferometer data of the Optical-RTM-derived sample; left: top, right: bottom surfaces; Figure S15: 2D view of the white light interferometer data of the standard RTM-derived sample—left: top, right: bottom surfaces; Figure S16: Force–displacement diagrams of the four-point bending tests of tGFRP plates 1–3 (top to bottom); Table S1: DSC results—24 h room temperature curing and 15 h tempering at 60 °C; Table S2: DSC results—24 h room temperature curing and 39 h tempering at 60 °C; Table S3: DSC results—24 h room temperature curing and 87 h tempering at 60 °C.
