# Supplementary material for: Method of Manufacturing Structural, Optically Transparent Glass Fiber-Reinforced Polymers (tGFRP) Using Infusion Techniques with Epoxy Resin Systems and E-Glass Fabrics
Source: Polymers (Basel). 2023 May 4;15(9):2183. doi: 10.3390/polym15092183 (PMC10180623; doi:10.3390/polym15092183)
Supplement: Supplementary file 1 [file polymers-15-02183-s001.zip › polymers-2303975-supplementary.pdf]

Supplementary

Method of Manufacturing Structural, Optically Transparent Glass Fiber-Reinforced Polymers (tGFRP) Using Infusion Techniques with Epoxy Resin Systems and E-Glass Fabrics

Klaus Heudorfer [1,†], Johannes Bauer [1,†], Yavuz Caydamli [2,3,†], Bruno Gompf [4], Jens Take [1], Michael R. Buchmeiser [2,3,*] and Peter Middendorf [1,*]

[1] Institute of Aircraft Design (IFB), University of Stuttgart, Pfaffenwaldring 31, D-70569 Stuttgart, Germany; heudorfer@ifb.uni-stuttgart.de (K.H.); bauer@ifb.uni-stuttgart.de (J.B.); take@ifb.uni-stuttgart.de (J.T.)

[2] Institute of Polymer Chemistry (IPOC), University of Stuttgart, Pfaffenwaldring 55, D-70569 Stuttgart, Germany; yavuz.caydamli@ipoc.uni-stuttgart.de

[3] German Institutes of Textile and Fiber Research (DITF), Körschtalstr 26, D-73770 Denkendorf, Germany

[4] 1st Physics Institute, University of Stuttgart, Pfaffenwaldring 57, D-70569 Stuttgart, Germany; b.gompf@pi.uni-stuttgart.de

* Correspondence: michael.buchmeiser@ipoc.uni-stuttgart.de (M.R.B.); middendorf@ifb.uni-stuttgart.de (P.M.)

† These authors contributed equally to this work.

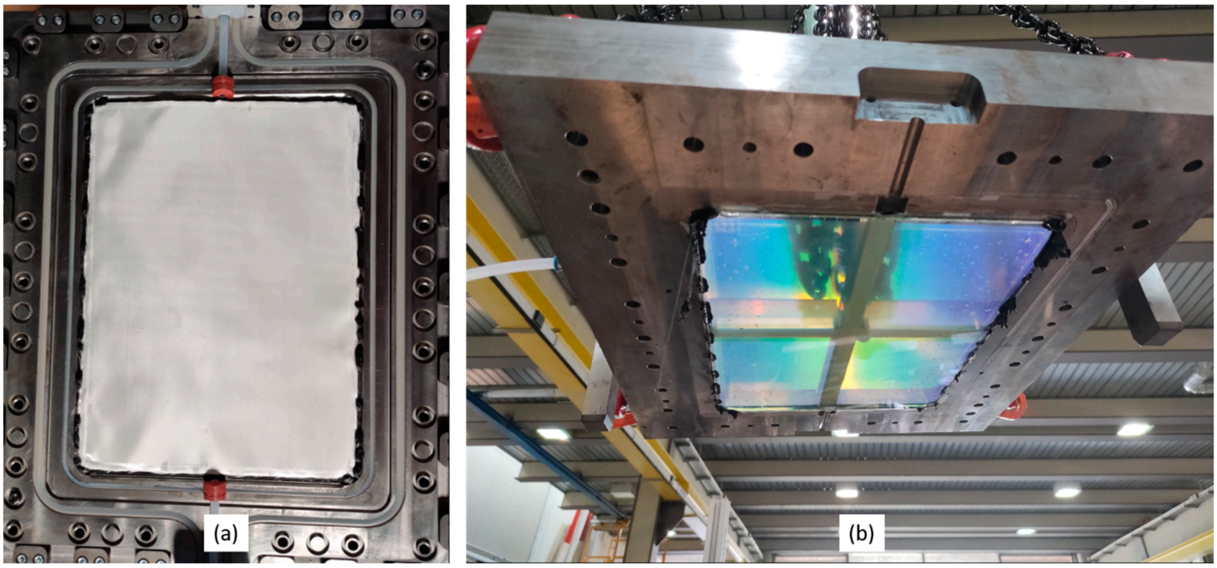

Figure S1. (**a**) Preform in the mold cavity and tacky tape (black) applied to prevent runners on the edges, (**b**) tGFRP plate sticking to the upper mold when opening after 24 h curing at room temperature.

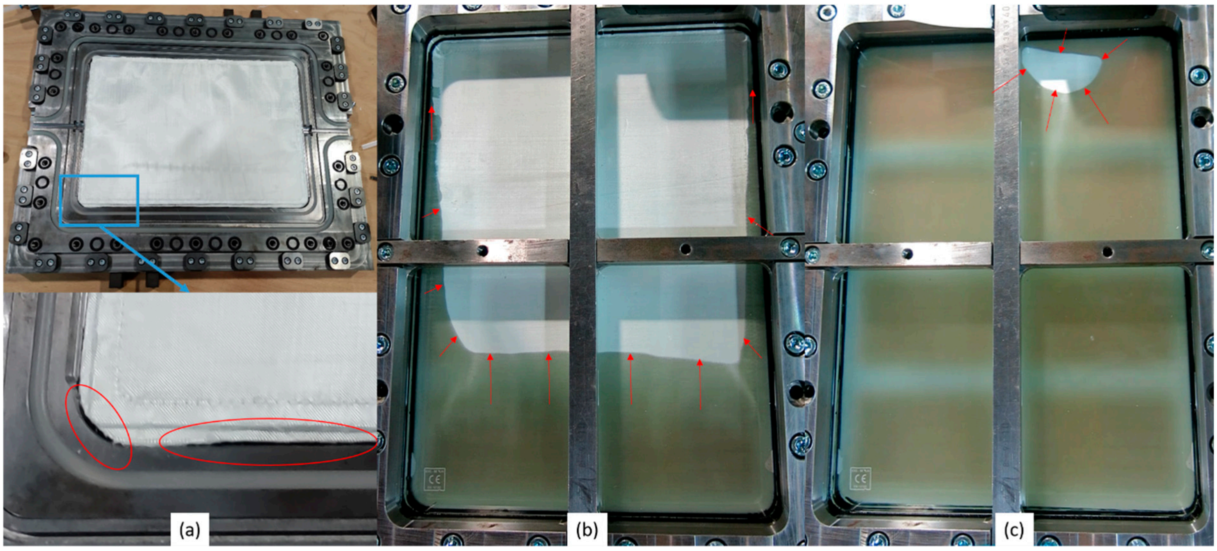

Figure S2. (**a**) Preform with cutting tolerances placed in a stiff mold with defined cavity dimensions leading to a fiber-free volume between preform and mold, (**b**) race-tracking (runners) on the preform edges, (**c**) uncontrolled flow-fronts colliding.

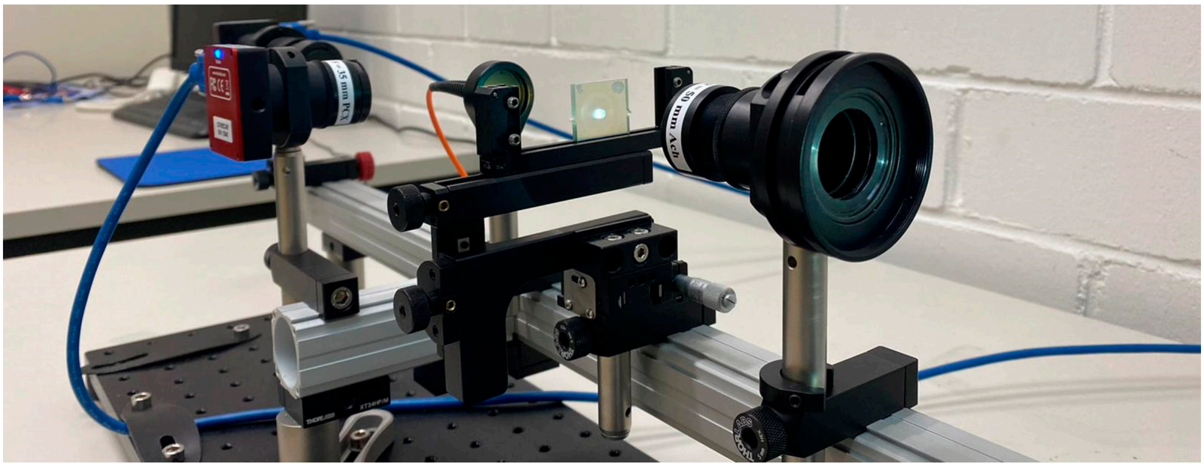

Figure S3. Spectrometer for transmittance measurements.

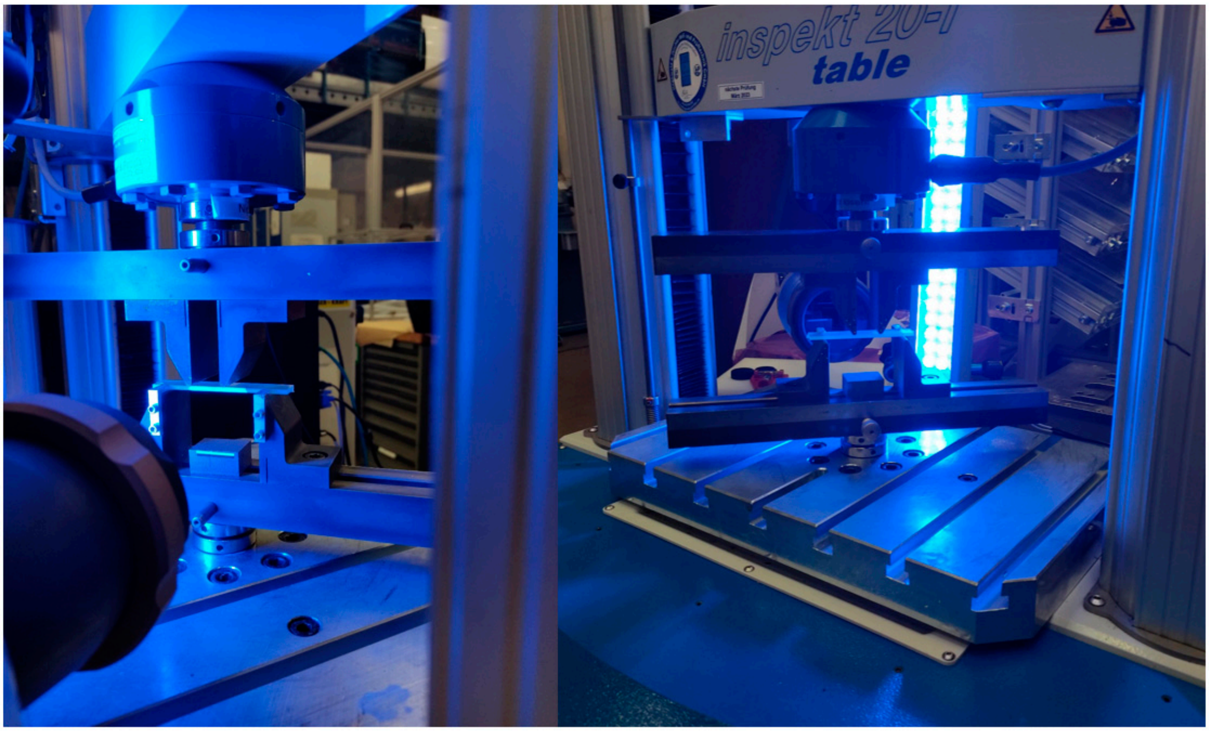

Figure S4. Four point bending apparatus (left: backside, right: front side).

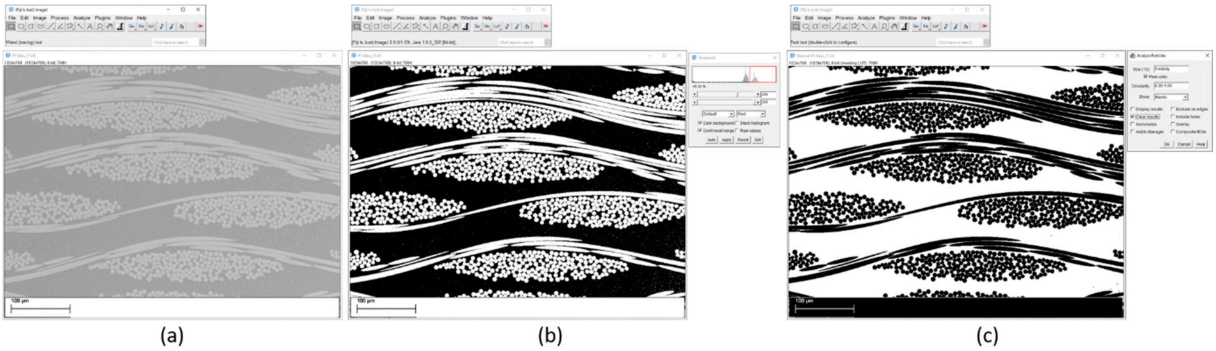

Figure S5. Image preprocessing using ImageJ: (**a**) 8-bit grayscale, (**b**) BW-image with threshold values, (**c**) mask of BW-image.

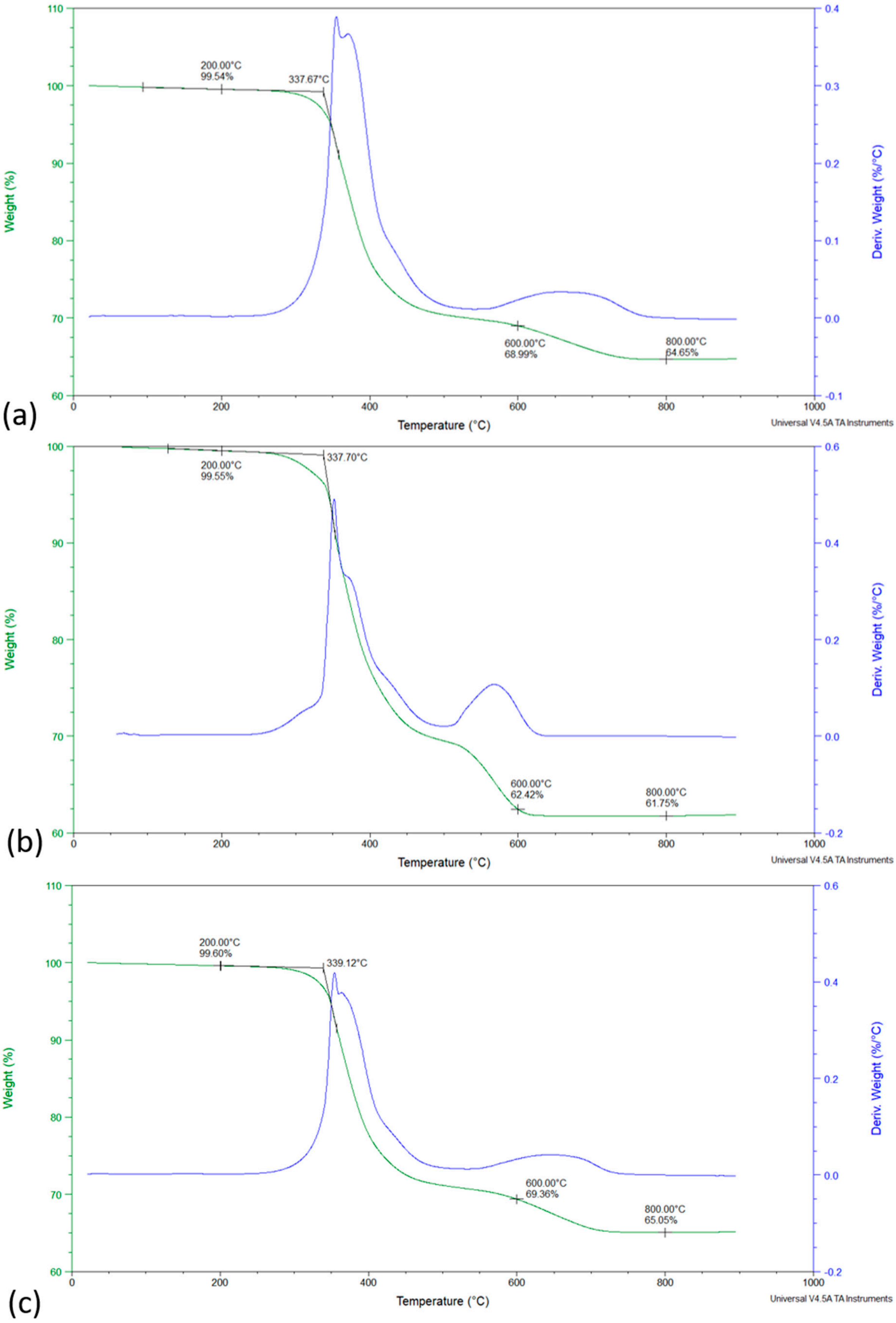

Figure S6. TGA Results of plate (**a**) 1, (**b**) 2 and (**c**) 3.

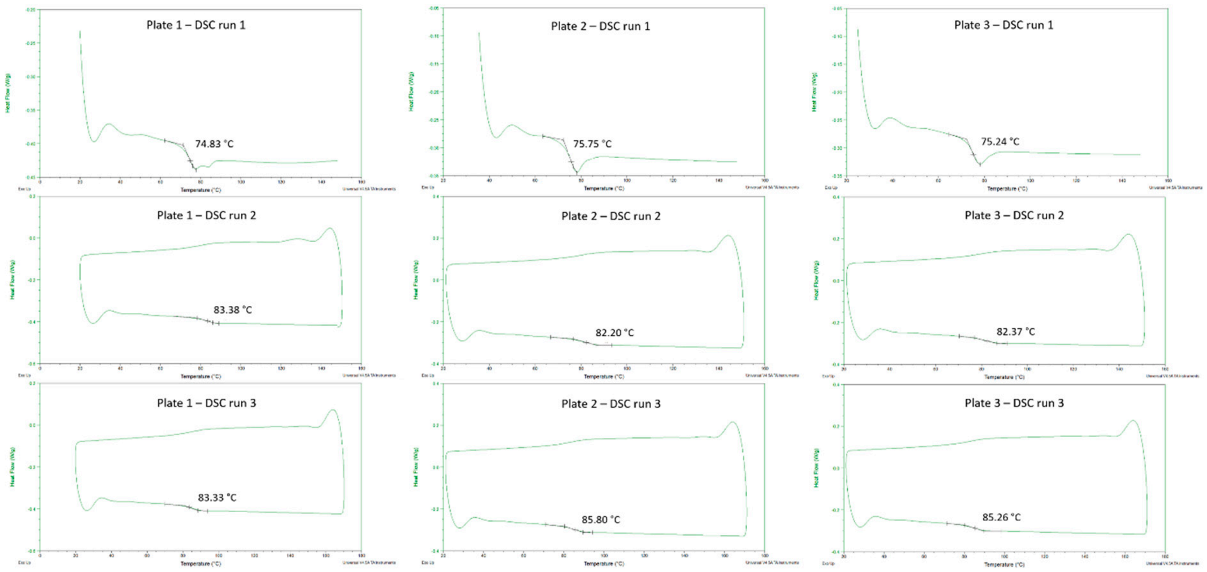

Figure S7. DSC results of tGFRP specimens after curing for 24 h at room temperature plus tempering for 15 h at 60 °C.

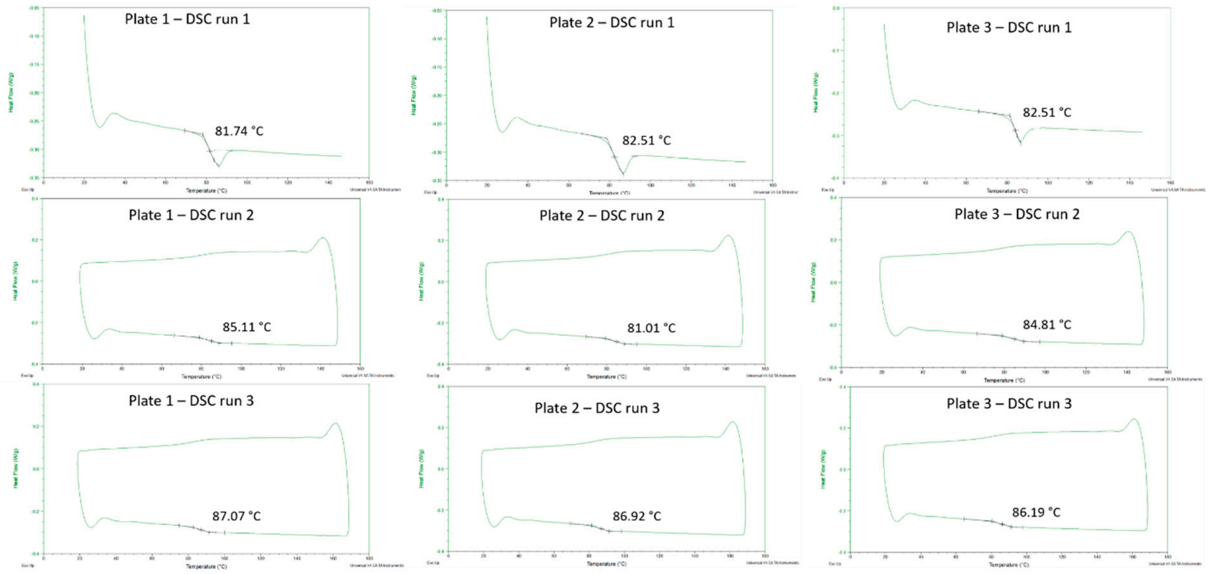

Figure S8. DSC results of tGFRP specimens after curing for 24 h at room temperature 39 h at 60 °C.

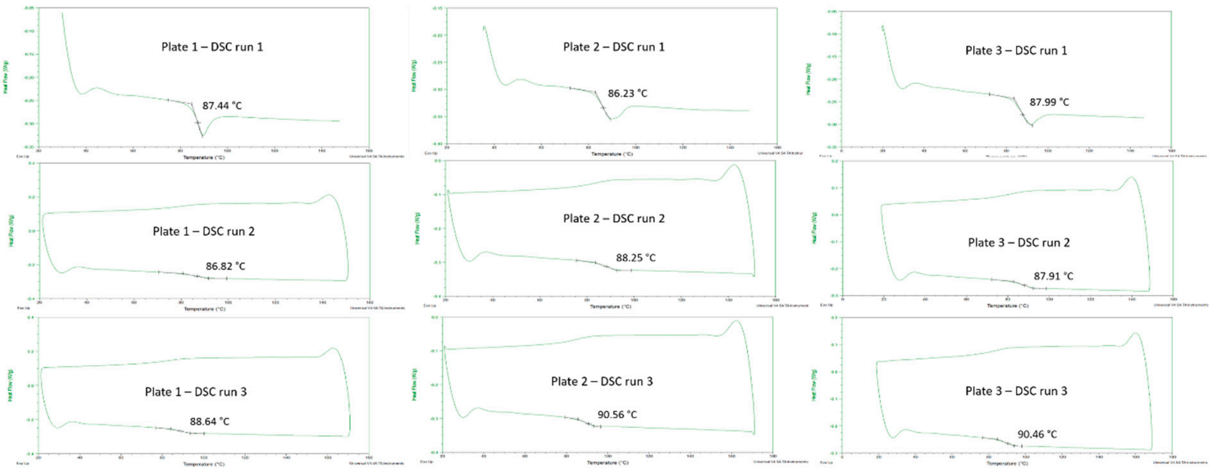

Figure S9. DSC results of tGFRP specimens after curing for 24 h at room temperature plus 87 h tempering at 60 °C.

Table S1. DSC results: 24 h at room temperature cure plus 15 h tempering at 60 °C.

tGFRP	Tg DSC run 1 [°C]	Tg DSC run 2 [°C]	Tg DSC run 3 [°C]
**Plate 1**	74.49	83.38	83.33
**Plate 2**	75.75	82.20	85.80
**Plate 3**	75.24	82.37	85.26
**Mean**	75.16	82.65	84.80
**Standard deviation**	0.63	0.64	1.30

Table S2. DSC results: 24 h at room temperature cure plus 39 h tempering at 60 °C.

tGFRP	Tg DSC run 1 [°C]	Tg DSC run 2 [°C]	Tg DSC run 3 [°C]
**Plate 1**	81.74	85,11	87,07
**Plate 2**	82.51	85,01	86,92
**Plate 3**	84.01	84,81	86,19
**Mean**	82.75	84.98	86.73
**Standard deviation**	1.15	0.15	0.47

Table S3. DSC results: 24 h at room temperature cure plus 87 h tempering at 60 °C.

tGFRP	Tg DSC run 1 [°C]	Tg DSC run 2 [°C]	Tg DSC run 3 [°C]
**Plate 1**	87.44	86.82	88.64
**Plate 2**	86.23	88.25	90.56
**Plate 3**	87.99	87.91	90.46
**Mean**	87.22	87.66	89.89
**Standard deviation**	0.90	0.75	1.08

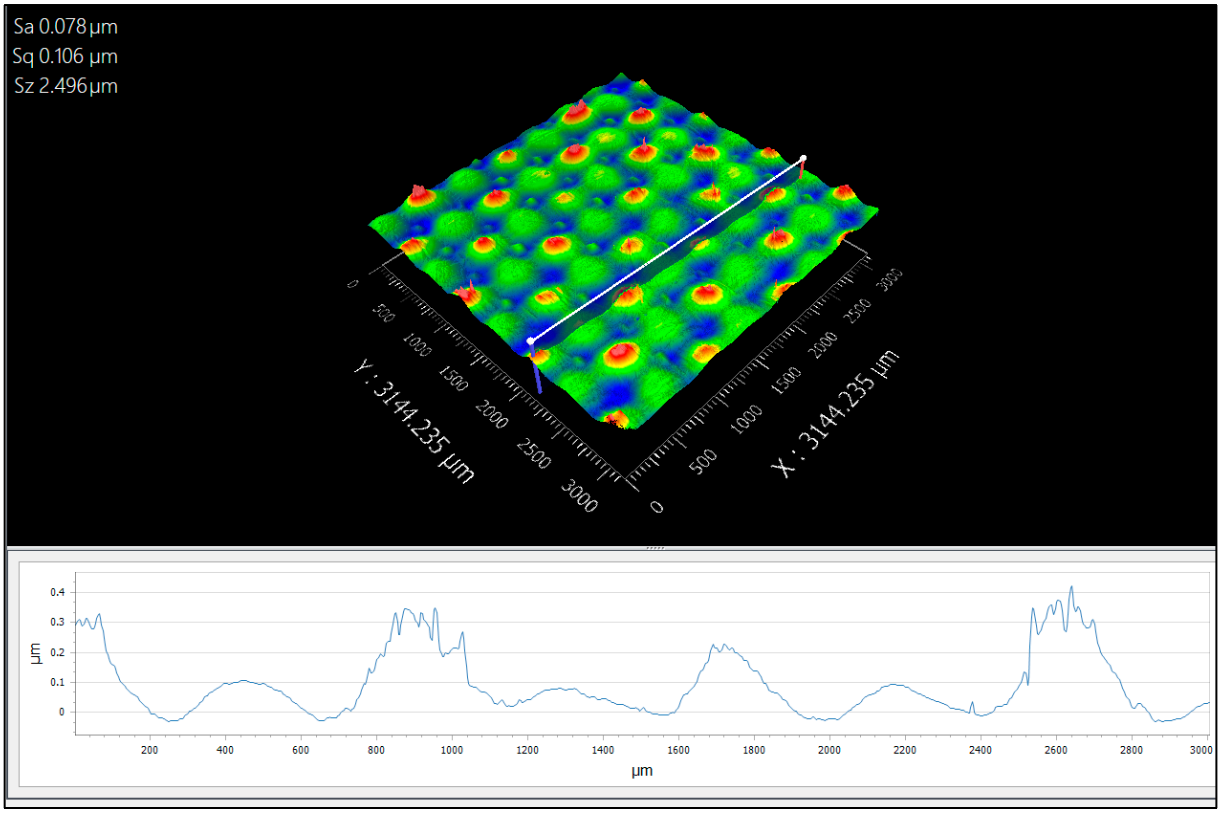

Figure S10. White light Interferometer data of the top surface of the Optical-RTM produced sample.

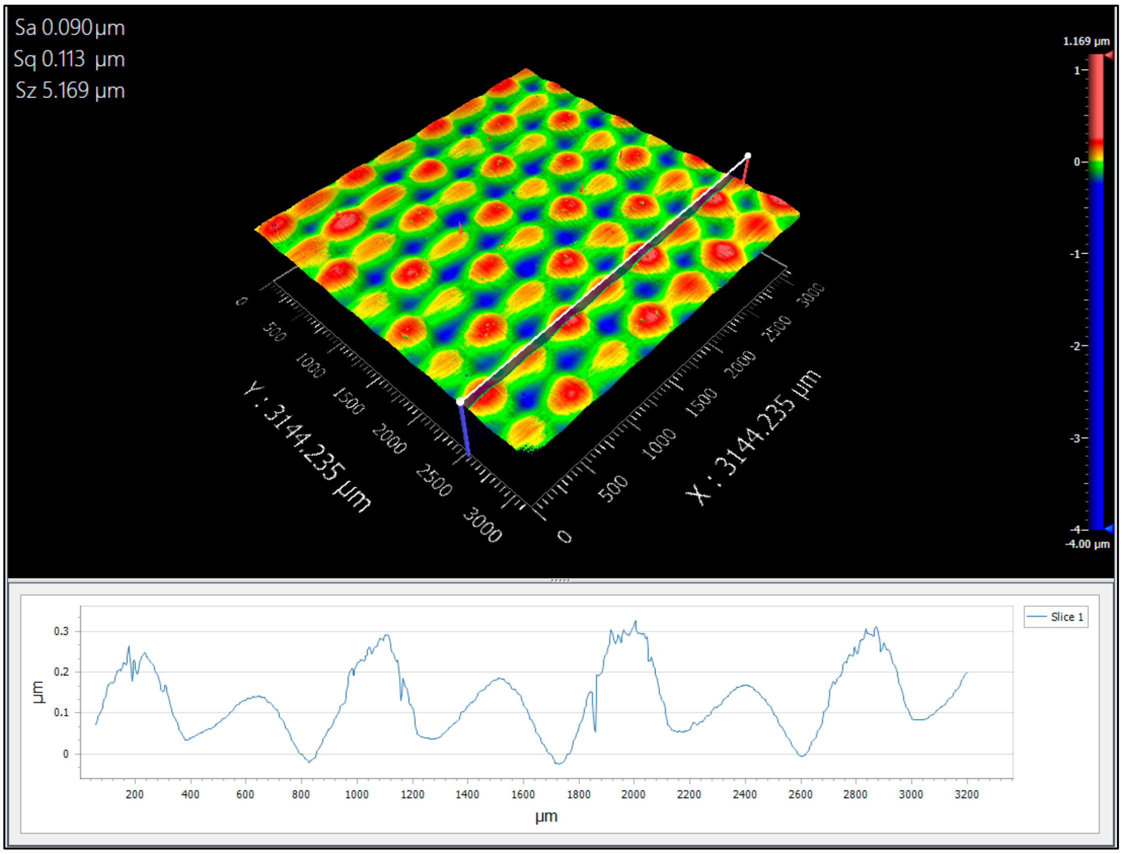

Figure S11. White light Interferometer data of the bottom surface of the Optical-RTM produced sample.

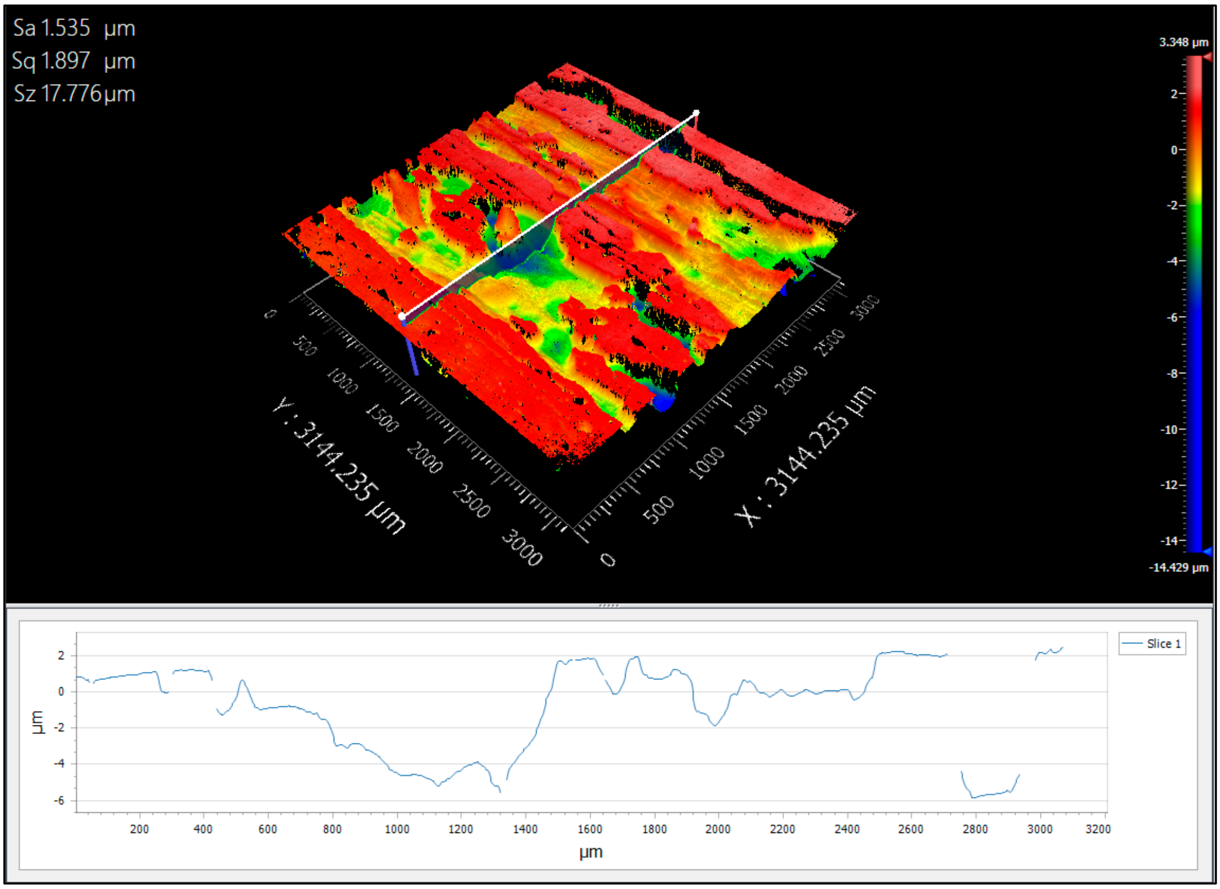

Figure S12. White light Interferometer data of the top surface of the standard-RTM produced sample.

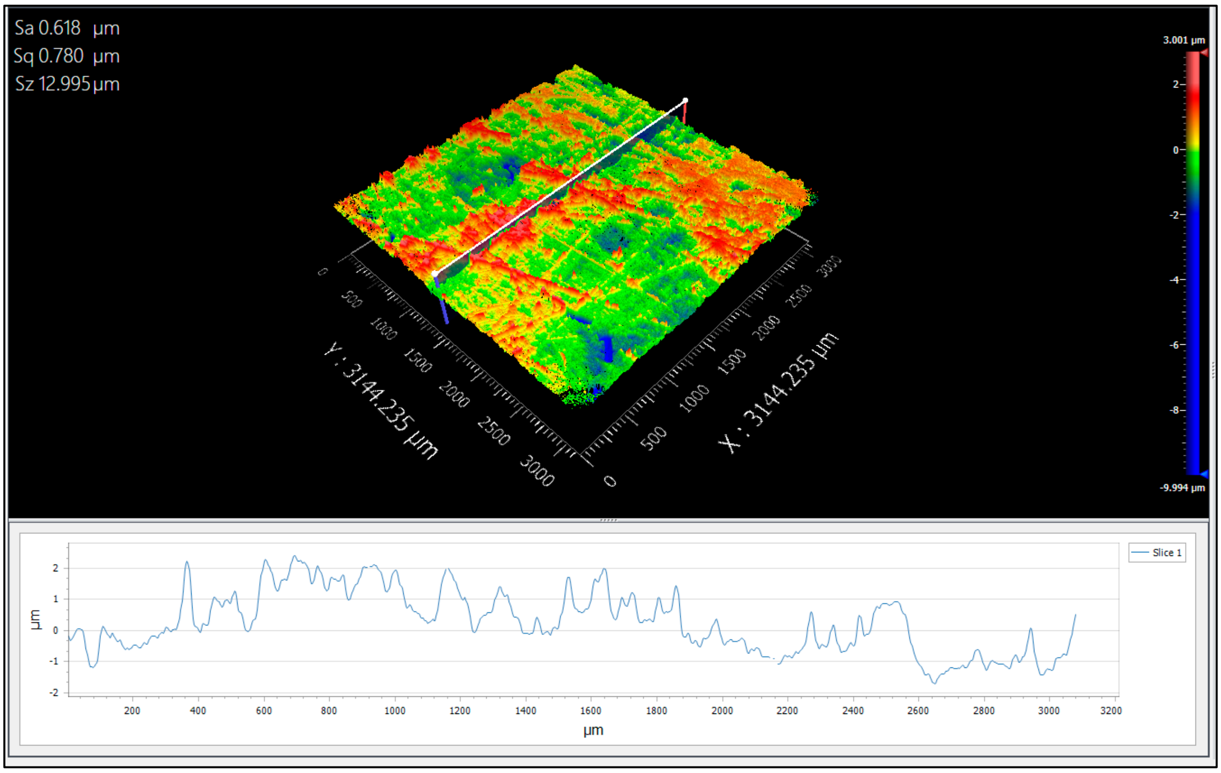

Figure S13. White light Interferometer data of the bottom surface of the standard-RTM produced sample.

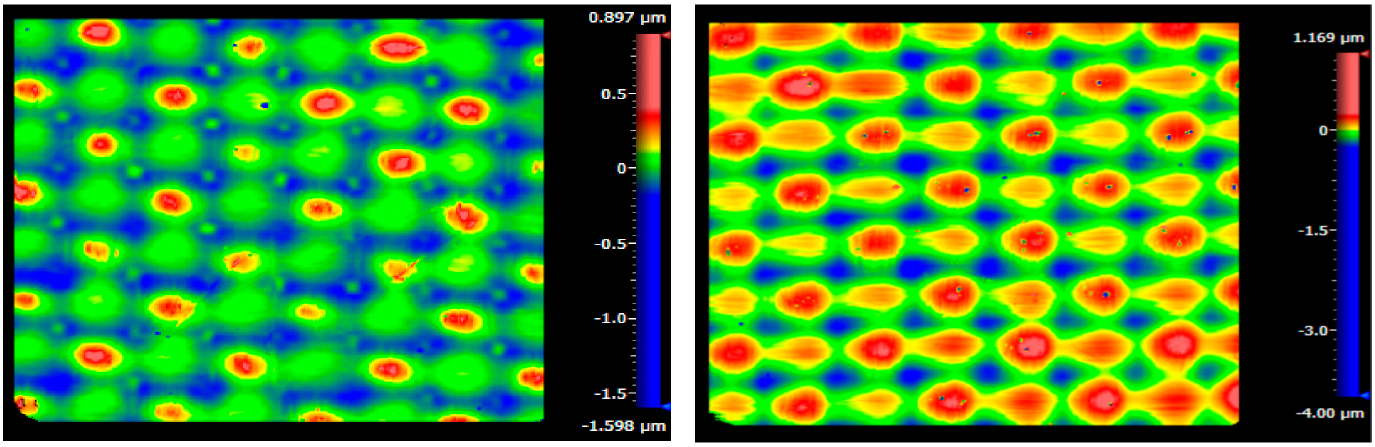

Figure S14. 2D view of the white light interferometer data of the Optical-RTM produced sample; left: top, right: bottom surfaces.

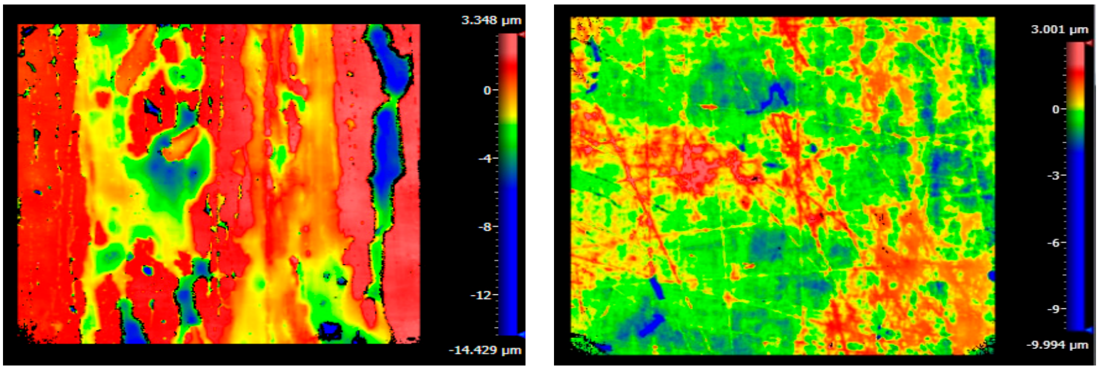

Figure S15. 2D view of the white light interferometer data of the standard-RTM produced sample; left: top, right: bottom surfaces.

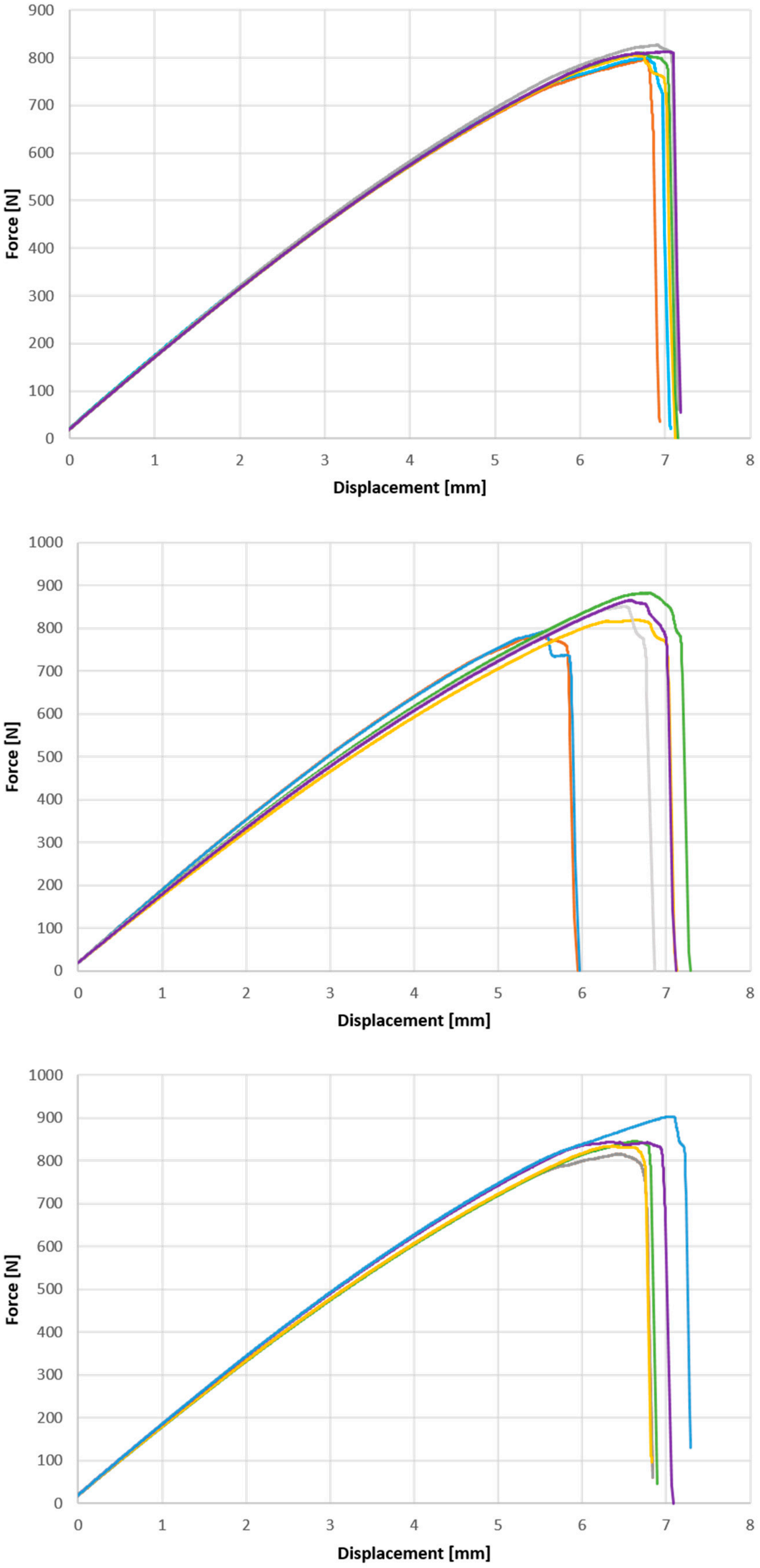

Figure S16. Force-Displacement diagrams of the four point bending characterization of tGFRP plate 1-3 (top to bottom).
